# A review of feto-placental vasculature flow modelling

**DOI:** 10.1016/j.placenta.2023.08.068

**Published:** 2023-10

**Authors:** Joanna Chappell, Rosalind Aughwane, Alys R. Clark, Sebastien Ourselin, Anna L. David, Andrew Melbourne

**Affiliations:** a School of Biomedical Engineering and Imaging Sciences (BMEIS), King's College, London, UK; b Elizabeth Garrett Anderson Institute for Women's Health, University College, London, UK; c Auckland Bioengineering Institute, New Zealand

**Keywords:** Placenta, Computational modelling, Placenta flow modelling, Placental haemodynamics

## Abstract

The placenta provides the vital nutrients and removal of waste products required for fetal growth and development. Understanding and quantifying the differences in structure and function between a normally functioning placenta compared to an abnormal placenta is vital to provide insights into the aetiology and treatment options for fetal growth restriction and other placental disorders. Computational modelling of blood flow in the placenta allows a new understanding of the placental circulation to be obtained. This structured review discusses multiple recent methods for placental vascular model development including analysis of the appearance of the placental vasculature and how placental haemodynamics may be simulated at multiple length scales.

## Highlights

• Many mathematical models and vascular scales exist within the literature.
• This review discusses multiple methods for placental vascular model development.
• Future developments are likely to bring these models in to in vivo use.

## 1 Introduction

**Fig. 1:**
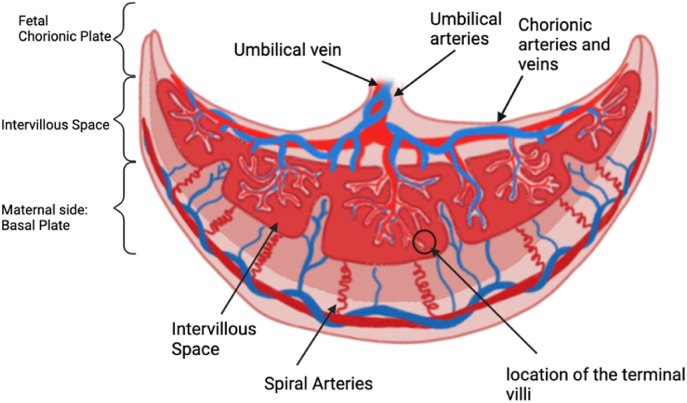
The anatomy of the human placenta identifying the different levels of the placenta broken down as the umbilical, large vessels, maternal and terminal in the review as well as the multiscale covering the combination of these levels.

The placenta provides essential exchange of nutrients and waste products between the mother and the fetus and is vital for healthy fetal growth and development [1]. The feto-placental vasculature is a complex branching network, beginning from the insertion of the umbilical arteries and branching into 6–8 generations of vessels which traverse over the whole chorionic plate of the placenta [2]. In humans, the two umbilical arteries deliver deoxygenated blood to the placental umbilical cord insertion at the chorionic plate and spread across the placental surface before branching into the stem villi which in turn branch into the mature immediate villi and finally the terminal villi; this is where the exchange of nutrients and waste products occur [1]. This is illustrated in Fig. 1. Fetal development is entirely dependent on placental function. Any impairment can result in health implications which can extend into adulthood [[3], [4], [5]].

The placenta is extraordinarily diverse between species. This makes it difficult to establish animal models that fully replicate the function of the human placenta [6]. Mammalian placentas are typically divided into canonical descriptions based upon their gross appearance and the number of cell layers between maternal and fetal circulations. Of note, the rodent placentas of mice, rats and guinea pigs come close to human placentas by having a similar vascular structure and a discoid appearance despite their smaller scale [7]. Sheep are an important model of human pregnancy and their size and resilience makes them amenable to validation studies of human-scale techniques [8,9]. However, although the gross function of the sheep placenta between sheep and humans is quite similar, the placental structure is not, with sheep forming discrete placentomes at defined points on the uterus, and fetal and maternal circulations comprising interdigitated capillary beds. Non-human primate placentas represent the closest possible models of the human placenta [10], but conditions such as placenta accreta and pre-eclampsia do not occur in most non-human primates since the human placenta appears to differ significantly even from our primate and great ape cousins. The exceptional diversity of the placenta across species, although interesting, means that the human placenta is an organ for which the most relevant models are those designed and developed in the human placenta.

The two umbilical arteries (UAs) carry the oxygen poor, and carbon dioxide rich fetal blood to the placenta. The umbilical vein returns the oxygen enriched blood from the placenta to the fetus [11]. Commonly the two umbilical arteries coil around the umbilical vein [[12], [13], [14]]. The chorionic plate vessels connect the fetal circulation via the umbilical cord to the placental circulation of the villous trees [15]. The common connection between the UAs in most placentas is known as Hyrtl anastomosis. It is hypothesised that it plays a role in the distribution of fetal blood flow to the placenta by equalising the blood pressure between the UAs [16]. The UAs are strong muscular vessels with a mean diameter of 4.2 mm at 40 weeks’ gestation [17,18]. The umbilical vein is approximately 30% larger in cross-sectional area (∼28 mm^2^ [12]) than the UAs combined, and, the velocity is half that of either artery, approximately 10–22 cm/s [17]. Commonly, the two UAs form what has been described mathematically as a cylindrical helix around the umbilical vein [[12], [13], [14]].

The feto-placental vasculature resides within villous trees. Mature intermediate villi are those which contain the narrowest muscularised arteries reaching the 20–40 μ m diameter range [19]. They serve as the junctional segments between the smallest stem villi and the terminal villi, differing from the stem villi due to a reduction of collagen fibres [20]. The terminal villi can be imaged ex vivo often as small as 1-2 μ m [21]. The terminal villi contain the feto-placental capillaries and are present for the maximal diffusional exchange [15]. The number of terminal villi is therefore correlated to the degree of oxygenation [19].

The maternal circulation is separate from the fetal circulation, residing in the uteroplacental vasculature made up of coiled vessels known as spiral arteries. They play a key role in the supply of nutrients to the placenta [22,23].

The haemodynamics of the placenta are unique, with the mass exchange of oxygen and nutrients accomplished largely by diffusion across thin membranes between two blood supplies (maternal and fetal). The blood flow properties of both supplies have an influence on the placental function. Differences in vessel branching, increased vascular resistances and changes to blood flow velocity and direction relative to the opposing circulation have an influence on the transfer of oxygen and nutrients between the two circulations [24,25]. Blood flow is thus correlated to the transport of oxygen and other solutes and hence it is vital to understand how changes in blood flow influence pregnancy complications and the effect that these have on the maternal and fetal health.

During pregnancy significant haemodynamic and metabolic changes occur for fetal growth. The inability to adapt to these changes can lead to hypertensive disorders in pregnancy such as hypertension, preeclampsia, gestational diabetes and preterm birth [26]. Placental insufficiency is associated with additional obstetric disorders [27]. This a major contributor to conditions such as fetal growth restriction (FGR), affecting 10–15% of pregnancies [[28], [29], [30], [31]] and in the UK leading to approximately two-thirds of stillbirths [32]. The lack of understanding of both typical and insufficient placental development means that it can be hard to predict the development of conditions and at present there is no treatment that can improve placental function, fetal growth and development [33]. In FGR placentas, there appears to be reduced villous branching and narrower vessels leading to a significant reduction in fetal blood flow and hence oxygen and nutrient exchange [1,34,35]. This and inadequate maternal spiral artery remodelling lead to poor placental development which can lead to chronic hypoxia [[30], [36]]. Placental hypoperfusion and ischemia are commonly caused by abnormally narrow vessels with poorly developed branching patterns [37,38]. The vascular resistance within the fetal compartments of the placenta should be low, allowing for forward flow to the umbilical arteries. In pregnancies with gestational diabetes, the terminal villi show several structural alterations such as an increased number of immature villi, and hypervascularity occurs [39,40].

In vivo assessment of the vascularisation of the placenta is often carried out using Doppler ultrasound to assess placental blood flow and vascular impedance [28,41]. The flow velocity and pulsatility index (ultrasound Doppler waveform indices derived from the ratio of systolic to diastolic flow velocities) of blood in the umbilical arteries can indicate resistance of the placental vasculature [42]. This in turn provides information on blood velocity throughout the cardiac cycle. Ultrasound provides low-cost quantification of blood flow dynamics. However, although current (Doppler) US resolution is useful clinically for observing flow in the large umbilical and uterine arteries, the resolution and viewing angles do not normally permit the measurement of flow within smaller uterine or placental vessels [43]. New technology studies, including that by Collins et al. [44] utilise 3D power Doppler (PD) to observe the differences in spiral arteries with small for gestational age babies. PD provides an estimate of the degree of vascularity in tissue, by estimating a fractional moving blood volume within the tissue of interest [45,46].

MRI can also provide new models measuring local variations in placental structure and function, and, provide blood flow and oxygenation metrics [32,47,48].

High spatial resolution can be achieved utilising micro-CT, photoacoustic imaging, confocal, super-resolution and electron microscopy, but these must be completed ex-vivo. Micro-CT enables detailed visualisation of the 3D anatomy of contrast filled vessels. Here there must be a trade-off between sample size and resolution. Microscopy methods enable 2D and 3D cellular level imaging. However, imaging depth is limited, and only small areas can be imaged to a subcellular level [43,49,50].

Computational models of the feto-placental circulation have been used to understand how changes to the placental circulation affect its function. These models typically simplify the geometry of the feto-placental vasculature and simulate haemodynamics within the system [1,51,52]. To identify, prevent and treat placental dysfunction will require an understanding of the factors which determine placental function; utilising mathematical modelling of the vasculature and simulating haemodynamics provide an effective way to achieve this [4,53]. This is a process which has been completed in other organs such as the liver, kidney and heart, providing the flow parameters to predict and show the modelling of transport of solutes, blood and valuable haemodynamics in hopes of further aiding clinicians [[54], [55], [56]].

Through a structured review, this work investigates the computational methods used to simulate the placental vasculature and discusses how the current limitations could be resolved in the future.

## 2 Literature search

**Fig. 2:**
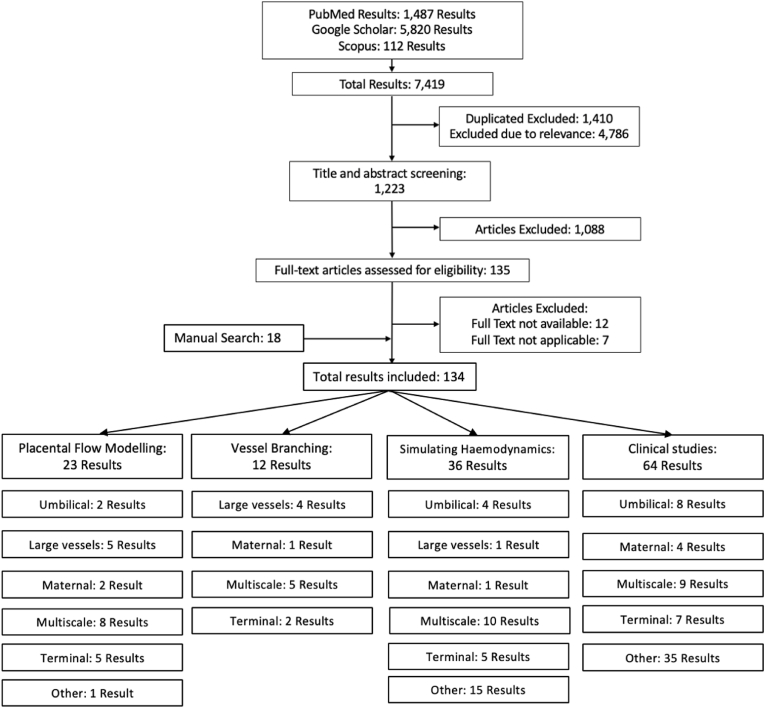
The flow diagram of the papers included and excluded in the review. Flowing down into 4 subcategories which were then split as the publications covering the umbilical cord, the top-level vessels such as the chorionic plate vessels, the maternal side of the placenta, multiscale models which cover all of the vessel levels of the placenta and the terminal microscopic level vasculature. Any other papers were categorised as other, the majority being general clinical placental publications.

A structured review was completed utilising PubMed, Google Scholar and Scopus. Placental Flow Modelling, Mathematical Flow Modelling and Simulating Placental Haemodynamics were all used as the key word entries. All non-English language papers and non-relevant titles/abstracts were excluded. Once the removal of repetition was completed 116 papers remained. Additional references from these papers were added manually reaching a total of 134 papers. The results of this process are shown in Fig. 2. Of the 134 journal articles identified, a total of 83 have been included in this review. The remaining 51 were omitted due to the content falling outside the scope of this review or overlapping with included studies.

Flow modelling of the placenta can be categorised into three subsections.

1. Large vessel modelling including the umbilical vessels and the chorionic vessels, often describing a less complex model of the blood flow. This macroscopic scale often relies on vascular corrosion casts and ex vivo CT to observe the vascular geometry.
2. Multiscale models which include the intermediate villi and the beginnings of the terminal villi, often utilising Micro-CT data.
3. Capillary level models which include the diffusion and movement of solutes between the vessels into the flow models, often supported using data from confocal laser scanning and electron microscopy.

## 3 Umbilical vessel and chorionic plate modelling

The top-level vasculature is associated with the chorionic plate (fetal) or basal plate (maternal) as seen in Fig. 1. On the fetal side, the vasculature begins with the umbilical cord insertion which branches down in the 6–8 generations of vasculature which traverse across the chorionic plate [2].

Models include that of Wen et al. [57] whose work aimed to assess the effect of spiralling in the human UA with pulsatile velocity waveforms. They assumed the blood to be isotropic and incompressible Newtonian fluid, with a constant dynamic viscosity. Then a pulsatile flow velocity waveform was used for the input and the flow analysis was completed with commercial computational fluid dynamics software. The average wall shear stress was calculated in the cardiac cycle as the instantaneous wall shear stress vector. The work did provide finite-element results as to the effect of the helical patterns of the umbilical vessels on the wall shear stress with different velocity waveforms. It was found that more coils enhanced the wall shear stress and that these differences can be further indicators of the differences in FGR umbilical arteries in terms of the reduction of coiling and reduced velocity profiles. This was an idealistic model due to the assumptions of blood flow modelling which does not take into consideration the potential effect of the connective tissue surrounding the umbilical cord known as the ‘Wharton's jelly’ which affects the torsional and compressive stresses imposed on the umbilical vessels and whether this would affect the results. The model was verified using ultrasound, but for further clinical utility would require in vitro laboratory experiments.

Kasiteropoulou et al. [58] focused on thermoregulation and the human maternal-fetal heat exchange of the umbilical cord (UC); their model predicted that the helical structure of the arteries plays a vital role in reducing blood temperature (and maintaining fetal temperature, as the fetus does not have the mechanisms for temperature control [59]). Fetal heat production and loss must remain balanced, if the umbilical circulation is affected externally, this can lead to temperature elevation potentially leading to hyperthermia where in severe cases the fetal growth and brain development could be affected. The model used the Navier-Stokes equations to model blood flow as steady, laminar and incompressible although the umbilical artery flow is more complex in reality [58].

The first order of chorionic plate arteries are 5–10 mm in length with an average diameter of 1.5 mm, with the accompanying vein being roughly 2 mm in diameter [60]. Gordon et al., 2007 [2] modelled the vasculature from a casting completed of an ex vivo perfused human placenta, taking the cast of the vessels and modelling the 3D geometry as basic branching units, to observe the effects of dichotomous and monopodial bifurcation. This was limited by the simplification of the cylinders used and the effect of the vasculature changing ex vivo. The work did show the ratio of each branch to the next and the decrease in diameter of the vasculature through 6–8 generations of vessel branches.

Guilot and Todros, 1992 synthetically modelled the initial insertion of the human umbilical arteries to the chorionic plate down multiple generations of branching without considering the distal fetal vessels. Their aim was to show the physical changes of the UA throughout pregnancy, without combining all vasculature together, but to model the individual vessel branching which had not been completed previously. Their flow models progressively branched into the chorionic vessels [61,62]. The structure mimicked a network of branches with a gradually reduced cross-sectional area and length, to 15–16 generations. This provided slightly larger 15th generation vasculature than those found in existing vascular castings [63] as well as accounting for the non-linear haemodynamic features. The models were limited by the prediction of resistances and effect on the flow and pressure of the joints. However, the results were novel in the flow modelling and vital for the continuation in the links between decreased flow and reduced branching networks of FGR.

**Fig. 3:**
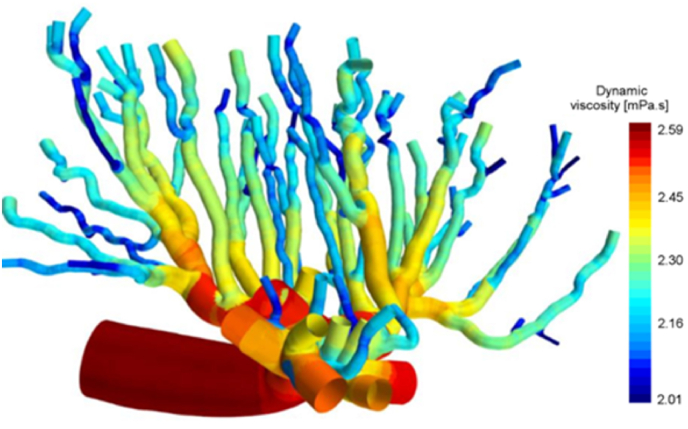
A sagittal fetal sub volume approximately 32% the size of the rat placenta 3D reconstructed from Micro-CT imaging modelling the dynamic viscosity reproduced with permission from Bappoo et al. Approximately 4.32 mm in length predicted from 32% of the placentas size [64].

Bappoo et al. [64] modelled the placenta from the Micro CT of a rat placenta reconstructing sections of the fetal chorionic vessels to model the viscosity (See Fig. 3). The haemodynamics utilised tested the effects on viscosity of different models, specifically the Newtonian, non-Newtonian and the Fåhræs-Lindqvist models. The viscosity results changed greatly between the models with the Newtonian model producing constant, absolute viscosity through the geometry. The non-Newtonian model was a more heterogeneous distribution and the Fåhræs-Lindqvist model the most comparable to viscosity values within literature. These results are limited by the correlation differences in structure between the human and rat placentas, alongside the effect of ex-vivo scanning. The full feto-placental network also could not be simulated due to the computational size of the model limiting the overall changes in the viscosity over the complete anatomy implying loss of haemodynamic detail.

The fetal blood in the placenta was assumed to be steady, incompressible and laminar for all of the models within this section, such that there was no resistance from the walls of the vessel. The Navier-Stokes equation was utilised to characterise blood flow which considers the velocity vector, fluid pressure, density and viscosity. Two equations, one of continuity enforcing the principles of mass and momentum and a second which enforces Newton's second law, were also used [5,53,65].

These umbilical and chorionic models were utilised to observe the effect of geometry and branching generations on the perfusion, viscosity, shear stress, temperature, velocity, pressure and flow profiles of the vessels. Splitting sections of modelling by the umbilical cord [57] and the larger vessel models [2,61,62] using vessel casting and Micro-CT. Utilising different haemodynamic models to model the blood and flow, has shown that the results for parameters such as viscosity can completely change with Bappoo's results showing a 100% difference in the viscosity between the Fåhræs-Lindqvist and Non-Newtonian models [64]. The models are, however, limited by only constructing the top-level vasculatures and not going further into exploring the solute transportation and terminal vessel branching of the placenta.

## 4 Multiscale modelling

Multiscale models extend the initial top level chorionic vessel to include the entirety of the chorionic plate and villous trees in fetal models and the intervillous space in maternal models. This includes the interaction between the macroscopic and microscopic scales [42].

Clark et al., 2015 [42] carried out multiscale modelling of a human canonical placenta from the initial umbilical cord insertion down to the fetal terminal vessels, which resulted into 92 villous trees covering 15 generations of vessel branches. This was novel in mathematically modelling the function of the placenta across spatial scales. The model assumed blood flow as a steady state and Poiseuille flow which changes, due to the resistance through the vessels. This model neglects the pulsatile nature of the blood flow, and the possible impacts of vascular branching on local flow patterns in the largest vessels. The model also looked at how the vessel structure and blood flow affect perfusion. The model's predictions of umbilical artery blood pressure and vascular resistances were all comparable to literature. In addition, the model indicated the effect that different locations in the umbilical cord insertion and placental shape have on perfusion and overall efficiency [42].

Tun et al. [37] studied the differences in shear stresses affecting the vessels between typical human and FGR placentas. They continued from the model by Clark simulating a smaller placenta and adding a 35% decrease in the vessel branching of FGR placentas. Endothelial cell shear stress was predicted computationally and at a macro and micro scale using a microfluidic shear stress culture system. The parameters for the macro and micro levels were population based. They were taken from literature from different placentas which may limit the effectiveness of the results. The work did conclude that the placental capillary shear stress increases in the computational models with FGR, with an increase in the shear stress and volumetric flow being three times higher in severe FGR in comparison to the normal placenta. They hypothesised that this may have implications for placental development, as elevated shear stress was shown to impede the random movement of endothelial cells that is required for vascular branching in cell culture [37].

**Fig. 4:**
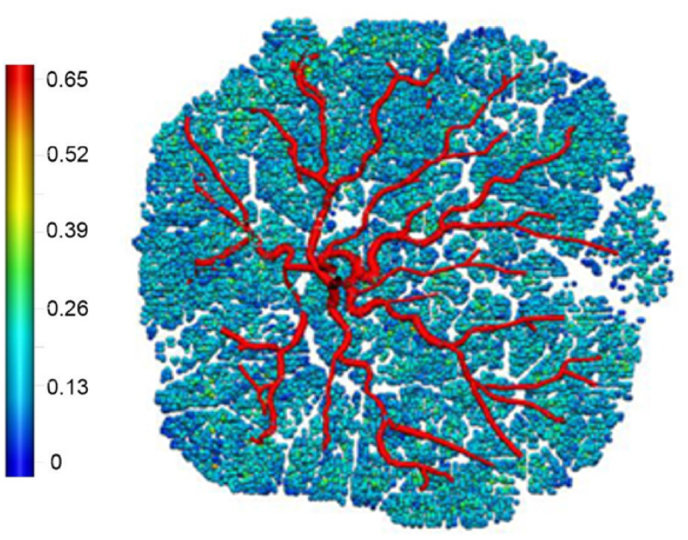
The multiscale flow model of an ex vivo human placenta from Micro CT quantifying the perfusion. The colour scale shows the volumetric flow in mm3/s reproduced with permission from Byrne et al. Approximate diameter of 20 cm [1].

Byrne et al. took the human fetal microscopic and macroscopic vasculature structures from perfused ex vivo placenta Micro CT images which were presented in patient specific computational models [1] (See Fig. 4). The computational model from Micro CT of the feto-placental vasculature was generated and the impact of the effect of vasculature on the total placental resistance and the blood flow heterogeneity observed. The haemodynamics assumed the blood flow to be in a steady state, incompressible and laminar. The effect of Hyrtl's anastomosis which is the common connection between the UAs near the cord insertion was observed and was speculated to equalise the blood pressure between the chorionic plate vessels [16]. This model was limited when the whole placenta was imaged using the micro-CT resolution, as it was not sufficient to capture every vascular branch, and, with the assumption that the blood flow was in a steady state rather than pulsatile. This showed a trend in increasing blood flow heterogeneity to relate to increase vascular resistance as well as the effect of Hyrtl's anastomosis to equalise the pressure between the regions perfused by each UA. The changes in local heterogeneity of the placenta were seen to lead to large increases in vascular resistance, such that local heterogeneity could be a predictor of increased risk in placental dysfunction [1].

Lin et al. [66] utilised a rule-based algorithm using length and diameter ratios from the literature through all the branch levels to reach 15 branches, representing a fetal villous tree. These parameters set the conditions such that there was sufficient space for each branch to grow. This led to a total of 202 terminal villi generated [66]. The maternal blood flow was modelled by Darcy's law and the pressure boundary conditions were fitted in the baseline flow velocity and a hydraulic conductivity coefficient set. The model outputs were the flow and pressure distributions of the maternal blood and rate of oxygen uptake by the villous tree. A placental oxygen exchange model was predicted and the oxygen concentration across the different vessel types and scales through the villous tree was changed. However, this was limited by the 2D placental cross-sectional slides which if extrapolated into a 3D model may lead to errors due to the lack of maternal and fetal blood flow. The placental oxygenation was sensitive to the vascular tree geometry as the tree must provide balance between maximising the surface area for gaseous exchange and allowing sufficient maternal blood flow [66].

Chernyavsky et al., 2010 [67] modelled streamlines of the input to the placenta from the human maternal spiral arteries, observing the key geometric and physical parameters of the ‘placentome’ simulating their effect on the transport of nutrients. The aim was to examine the impact of different vessel positions and the flow rates depending on geometric parameters of the placentome. The intervillous perfusion was modelled by an incompressible Newtonian liquid of viscosity μ. The ‘placentome’ was assumed to be enclosed in an impermeable hemisphere with a central source of a spiral artery [68]. Supplying blood at a steady rate which is asymmetric, the model assumed that the maternal blood flow is described by Darcy's law; as such the average blood velocity is proportional to the local pressure gradient, which is also proportional to the local pressure gradient of maternal blood [69]. Darcy's equation enables the intervillous blood pressure to be calculated. Visualisation of the vessels came from radioangiography of primate and human uteruses and the estimates of the villous volume fraction were based on stereological data. Experimental verification of the model by an ex vivo perfusion model and new techniques of ultrasound to further monitor changes in placental function would be required for further validation. The model was limited by its assumptions of the geometry and modelling of the blood flow, including not accounting for the fetal placental circulation and diffusion due to molecular motion. Although it is advantageous the simple structure and physiology of the maternal vasculature allows for the mathematical analysis which can be developed into more advanced models [67].

By displaying multiple scales of the placenta from the macro to micro haemodynamics, the models have provided an understanding in differing geometry. These include: the changing of the umbilical cord insertion, the placental shape and the vasculature branching generations and their effects on the pressure, the resistance, the blood flow, the shear stress and the placental oxygenation deeper in the placental tissue, through to 15 vessel branching generations. The models displayed both the typical placenta and the effect due to FGR, showing significant differences in the macro vasculature resistance and flow which is important for the continued growth in understanding of the differences in placental vasculature with FGR [70].The main limitations are due to the use of synthetic modelling of the vasculature, assumptions of constant flow, and ex vivo imaging as the changes in vasculature post-delivery may influence the outputted results [1]. The future of multiscale modelling is to further explore the gas exchange in 3D, with the influence of pulsatile flow and solute transfer [42].

## 5 Capillary level modelling

The deepest level of modelling is at the capillary level where gaseous exchange is occurring. Chorionic vessels branch into 60–100 villous trees covering on average 15 generations of vessel branches [42] At the ends of each of these villous trees are terminal villi. Confocal laser scanning is utilised to observe the geometry and velocity profiles of the vessels and Darcy's law used to model the flow as well as the inclusion of solute transport [71]. The computational size of the models is reduced due to models only considering a couple of the terminal villi. They are, however, vital in visualising the differences in terminal villi geometry in abnormal placentas and the differences that occur in the solute transfer [72].

Plitman Mayo et al. [21] scanned small human tissue samples containing terminal vessels that were from the computational modelling of the structure-function Relationship in human placental terminal villi. 3D blood flow and the oxygen transport in the human placenta were modelled and included flow validation from a plastic replica of the selected geometry. It was limited by lack of data regarding material properties of the vessels and a small sample time limits the accuracy of the model. However, the model did show that varying the capillary diameter is key for effective oxygen uptake by the fetus, displaying the advantages of combining immunofluorescent imaging and finite element analysis to display these effects.

Plitman Mayo et al. [73] were motivated to successfully model the oxygen diffusion transport of the human terminal villi in a 3D model of four different placental terminal villi specimens from reconstructions of confocal laser scanning microscopic image stacks. These specimens provided spatial arrangement of the fetal capillaries inside the terminal villi calculating the surface volume ratios and their effects on oxygen diffusion, which were comparable to the existing literature. This model was limited due to the mathematical description of the oxygen transport problem and the use of ex vivo imaging [73]. The requirement is for further validation of the model and improvement of the FE models to be more anatomically realistic.

**Fig. 5:**
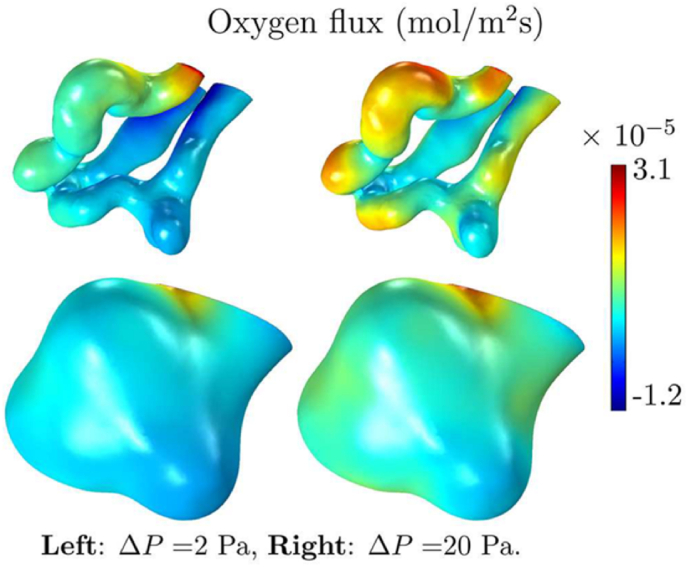
Modelling the human terminal villi from confocal laser scanning microscopy, showing the distribution of oxygen flux entering through the capillary at the top and the villous surface at the bottom. Reproduced with permission from Pearce et al. Approximate scale of 30–50 μ m [74].

3D simulation of the blood flow and oxygen transfer in human feto-placental capillaries [74] was combined with 3D simulations utilising the capillary surfaces from the laser scanning confocal microscopy by Pearce et al. (See Fig. 5). This work took place to quantify the effects of changing the geometry of the villi and the effect on oxygen transfer. Scaling the total oxygen transfer to the vascular resistance, the blood flow was modelled with Stokes equations and assuming non-Newtonian effects of red blood cells on the flow. However, the model was limited by vascular resistance overestimations and errors were introduced when converting the 3D geometries to the finite-element meshes. Despite this, the model displayed the relationship between the total oxygen transfer rate and the pressure drop through the capillaries, supporting the hypothesis that dilations of the capillaries enhance oxygen transfer without the requirement for extensive placental growth or remodelling. This was shown through the use of a regression equation which was used to estimate the oxygen transfer using the vascular resistance.

The effect of intervillous flow on solute transfer based on 3D imaging of the human placental microstructure was modelled by Perazzolo et al. [71] They were motivated to evaluate the effects of the maternal blood flow on the placental transfer of solutes and the effect different flow rates have on uptake. A 3D solid was reconstructed consisting of three main domains: intervillous space, villous barrier, and the fetal vessels from confocal laser scanning. The model provides a quantitative method to assess placental transfer of a generic diffusive solute. Modelling the blood as Newtonian and incompressible, the fluid flow regime was modelled as Stokes flow and the hydraulic permeability estimated by Darcy's Law. Assumptions as to the binding kinetics, efficiency of transfer and differences in sample structure led to considerable variability in the results. The model parameters were based on oxygen due to its small size. However, it would be expected that larger molecules could be incorporated. The study highlighted the importance of the local maternal flow motions in the placental microstructure for solute transfer.

Erlich et al. went on to further estimate the solute transfer within the human terminal villi, building from the above models with additional dimensionless parameters to model diffusion-limited transport [25,75].

Modelling the capillary level vessels is important for the understanding of the different effects placental micro vasculature has on solute transport in particular the oxygen transfer; such that the villi size and the oxygen transfer are positively correlated, and the increased vascular resistance decreases the oxygen transfer. These are the same conclusions as those drawn from the multiscale models. All models utilised ex vivo imaging, confocal laser imaging, of the capillary levels. This is limited by changes in the vasculature ex vivo when compared to in vivo, due to the reduced vessel lumen size [1,76]. However, having in vivo imaging to the resolution of terminal villi would require huge leaps in the current technology. These models could have applications in FGR where it is predicted that terminal villi geometry and growth reduces nutrient transfer to the growing fetus [77,78].

## 6 Additional considerations

The majority of the models reviewed here focused on representing the geometry of the levels of the feto-placental vasculature at delivery, as most used ex vivo imaging of the placenta. However, future considerations to mathematical modelling of the placenta could include modelling at different points across gestation, utilising in vivo medical imaging, observing comparisons in modelling of other maternal and fetal anatomy such as the cardiovascular systems [79] as well as different animal models of the placenta [80].

The placental vasculature develops across gestation and anomalies in placental development can arise very early in gestation, being found within the initial trophoblast cells [81]. Future considerations as to the structural changes of the placenta over gestation and how these can inform potential clinical interventions will be important for further understanding [42]. As the placenta is inaccessible to measurement, it is likely that in vivo data will be required to develop computational models of early gestation. Future improvements in MRI modelling and reconstruction should provide additional in-vivo imaging of the placenta. Models such as IVIM and DECIDE to observe the perfusion of the placenta between normal and abnormal pregnancies and quantification of placental function during gestation could be utilised to parameterise or validate models [32,47,82].

Unlike most other organs, the human placenta is different from other animal placental systems with substantive variations in the placental anatomy [42]. This provides difficulty in terms of relating animal models to human. However, within animal studies there is greater ease in influencing the growth of the placenta, and, measuring and changing the oxygenation to the placenta invasively during pregnancy [48]. Within animal fetal surgery there can be catheters implanted into the fetal arteries to measure the blood oxygenation. These practices are not possible within human studies, but provide important clinical verification of models within pregnant animals [9].

Models could potentially be devised to model individual fetal organs or to model the whole fetal system where the effect of placental dysfunction on the heart and organ development of the fetus can be directly coupled in a model [ [79,83,84]]. This would add complexity to existing models, but could increase understanding of clinical data. Translating these models into clinical use would require high speed and accuracy in relation to available clinical data, such that the patient specific model will be useful for treatment planning [44,45].

## 7 Conclusion

This structured review of the mathematical modelling of feto-placental vasculature aimed to summarise the variety of mathematical models and vascular scales that have been completed within existing literature. There is incredible complexity within the placenta and all the factors required to mathematically model the placenta with clinical accuracy. These covered the umbilical vessels, the maternal vessels, large chorionic plate vasculature, and the connective branching all the way through 15 generations of vascular branching to the terminal villi.

From the review, the multiscale modelling from ex vivo imaging showed the most promise in predicting function at multiple spatial scales. The capillary level modelling shows promise with the inclusion of the effect of nutrient transport and exchange, which is critical to determining the health of a pregnancy. Combining these for a more complex multiscale model would be very interesting future work. Taking these models and applying them to models of placental insufficiency could have a direct clinical utility by helping to understand the haemodynamic changes in the placenta for conditions such as FGR.

The verification of mathematical models for future clinical use is vital. This will include the quantification of changes between the in vivo and ex vivo placenta, alongside the matching of multiscale imaging such that all levels of branching can be parameterised by medical imaging. New opportunities for incorporating in vivo data form advanced MRI or Ultrasound will support the further development of these models and bring them closer to clinical translation.

## CRediT authorship contribution statement

J.Chappell: Conceptualization, Writing-Original draft. R.Aughwane: Writing- Reviewing and Editing. A.R.Clark: Writing-Reviewing and Editing. A.L.David: Writing-Reviewing and Editing. A.Melbourne: Supervision, Writing-Reviewing and Editing.

## Declaration of competing interest

The authors declare that they have no known competing financial interests or personal relationships that could have appeared to influence the work reported in this paper.
